# The Epithelial-to-Mesenchymal Transition-Like Process Induced by TGF-β1 Enhances Rubella Virus Binding and Infection in A549 Cells via the Smad Pathway

**DOI:** 10.3390/microorganisms9030662

**Published:** 2021-03-23

**Authors:** Ngan Thi Kim Pham, Quang Duy Trinh, Kazuhide Takada, Chika Takano, Mari Sasano, Shoko Okitsu, Hiroshi Ushijima, Shihoko Komine-Aizawa, Satoshi Hayakawa

**Affiliations:** 1 Division of Microbiology, Department of Pathology and Microbiology, Nihon University School of Medicine, Tokyo 173-8610, Japan; pham.thikimngan@nihon-u.ac.jp (N.T.K.P.); takada.kazuhide@nihon-u.ac.jp (K.T.); takano.chika@nihon-u.ac.jp (C.T.); shoko-tky@umin.ac.jp (S.O.); ushijima-hiroshi@jcom.home.ne.jp (H.U.); aizawa.shihoko@nihon-u.ac.jp (S.K.-A.); 2 Department of Neurological Surgery, Nihon University School of Medicine, Tokyo 173-8610, Japan; sasano.mari@nihon-u.ac.jp

**Keywords:** rubella, TGF-β1, virus binding, infection, EMT, smad, lung, cytometric analysis

## Abstract

Virus–host cell interactions in rubella virus (RuV) are of great interest in current research in the field, as their mechanism is not yet well understood. By hypothesizing that the epithelial-to-mesenchymal transition (EMT) may play a role in RuV infection, this study aimed to investigate the influence of TGF-β1-induced EMT of human lung epithelial A549 cells on the infectivity of RuV. A549 cells were cultured and treated with TGF-β1 for 1 to 2 days prior to virus infection (with a clinical strain). Viral infectivity was determined by flow cytometry analysis of cells harvested at 24 and 48 h post-infection (hpi) and by titration of supernatants collected at 48 hpi. The results showed that the percentages of the TGF-β1-treated A549 cells that were positive for RuV were at least twofold higher than those of the control, and the viral progeny titers in the supernatants collected at 48 hpi were significantly higher in the treatment group than in the control group. In addition, the virus binding assay showed a strong increase (more than threefold) in the percentages of RuV-positive cells, as determined by flow cytometry analysis and further confirmed by real-time PCR. Such an enhancement effect on RuV infectivity was abolished using LY364947 or SB431542, inhibitors of the TGF-β/Smad signaling pathway. The findings suggest that the TGF-β1-induced EMT-like process enhances RuV binding and infection in A549 cells via the Smad pathway. Further studies are necessary to identify possible proteins that facilitate viral binding and entry into treated cells.

## 1. Introduction

Rubella virus (RuV), which belongs to the genus *Rubivirus* in the family Matonaviridae [1], causes systemic infection in children and young adults and congenital rubella syndrome in developing fetuses. Virus–host cell interaction in RuV is of great interest in current research in the field, as its mechanism is not yet well understood. Regarding virus entry, myelin oligodendrocyte glycoprotein (MOG) is known to be a cellular receptor for RuV [2]; however, it is mainly expressed in the central nervous system. A Ca^2+^-dependent mechanism observed in lymphoid cells, involving a direct interaction between RuV E1 protein and sphingomyelin/cholesterol-enriched membranes, and a Ca^2+^-independent mechanism involving an unidentified RuV receptor have recently been reported [3] and require further investigation. In addition, factors affecting virus binding and infection are not well recognized.

Epithelial-to-mesenchymal transition (EMT) is a process by which differentiated epithelial cells develop a mesenchymal phenotype with migratory and invasive potential. EMT not only plays an important role in embryonic organogenesis but also is involved in wound healing, tissue regeneration, fibrosis and cancer progression (reviewed in refs) [4,5]. Recent findings suggest that there may be a reciprocal interaction between EMT and some viruses. Viral infection caused by rhinovirus, hepatitis C virus or Epstein–Barr virus could promote EMT [6,7,8]. In turn, EMT could abolish the susceptibility of epithelial cells to some viruses, such as the measles virus [9].

By hypothesizing that EMT may play a role in RuV infection [10], in this study, we investigated the influence of the EMT process in lung epithelial cells on RuV infection, a pathophysiological change associated with fibrosis in the context of chronic lung diseases, including asthma, idiopathic pulmonary fibrosis (IPF) and chronic obstructive pulmonary disease (COPD) [11,12,13]. By utilizing A549 cells as an in vitro model of lung epithelial cells and inducing experimental EMT via Transforming Growth Factor (TGF)-β1, as previously described [14,15], we found that the EMT-like process induced by TGF-β1 could enhance RuV binding and infection in A549 cells.

## 2. Materials and Methods

### 2.1. Cell Culture and Virus

A549 and Vero cells were purchased from the Japanese Collection of Research Bioresources Cell Bank and were cultured in Dulbecco’s modified Eagle’s medium (DMEM) (Gibco-Invitrogen, Tokyo, Japan). The cells were cultured in MEM (Gibco-Invitrogen, Tokyo, Japan). The above media were supplemented with 10% fetal bovine serum (FBS), 100 IU/mL penicillin and 100 μg/mL streptomycin. The cells were cultured in monolayers at 37 °C in a humidified 5% CO_2_ incubator.

The clinical RuV strain (3-B1-RK13) was obtained from Kitasato University School of Medicine (Tokyo, Japan). The virus stock solution was prepared by propagating the virus in Vero cells and then concentrating the viral particles by ultracentrifugation at 52,000× *g* for 90 min in the S50A rotor of a Himac CS100GX micro-ultracentrifuge (Hitachi Koki Co., Ltd., Ibaraki, Japan). Viral titers were estimated by the 50% cell culture infectious dose (CCID50) method, or by flow cytometry (FCM) analysis, as briefly described below.

### 2.2. Titration of the Virus Solution by FCM Analysis

To determine the viral titers of the concentrated virus stock solutions or collected supernatants during the experiments, a 30-μL volume of serial threefold dilutions of the virus solution was used to infect (in duplicate) freshly seeded Vero cells in a 96-well plate (4 × 10^4^ cells/well). Medium containing 2% FBS and NH4Cl was added at 6 hpi to prevent a second round of infection (final concentration of NH4Cl, 20 mM). At 24 hpi, the cells were collected and subjected to intracellular staining of the RuV capsid protein. Viral titers (in infectious units (IUs)) were then determined by FCM analysis. The virus titer in a sample was calculated as the average of 3 titers measured in 3 consecutive wells with a percentage of RuV-infected cells lower than 40% and higher than 0.3%, as described elsewhere [16].

### 2.3. Epithelial-to-Mesenchymal Transition Induced by TGF-β1 Treatment

A549 cells were seeded onto 6-well plates (2 × 10^5^ cells/well) or 96-well plates (10^4^ cells/well) in DMEM/10% FBS, and experiments were performed when a monolayer of the cells reached 70–80% confluence. The culture medium was replaced with serum-free DMEM containing 0.1% bovine serum albumin (BSA) for 2 h prior to stimulation with recombinant TGF-β1 (10 ng/mL) (Abcam, Cambridge, UK) in DMEM/0.1% BSA for 24 to 48 h. The cells cultured in serum-free DMEM/0.1% BSA were used as mock-treated (control) cells. The old medium was replaced with freshly prepared medium with or without TGF-β1 after 24 h.

For investigation into the roles of TGF-β1 signaling pathways in RuV infection, the A549 cells were incubated with one of the following inhibitors: SB203580 (20 μM, an inhibitor of the p38 mitogen-activated protein kinase (MARK) signaling pathway, Invitrogen), U0126 (25 μM, an inhibitor of the mitogen-activated protein/extracellular signal-regulated kinase kinase (MEK)/ERK1/2 signaling, Invitrogen), LY364947 (18 nM, a selective inhibitor of TGF-β type I receptor, Abcam), SB431542 (10 μM, an inhibitor of the TGF-β/Smad signaling) (Cayman Chemical, Ann Arbor, MI, USA) and SP600125 (50 μM, a c-Jun N-terminal Kinase (JNK) selective inhibitor, Abcam) for 1 h prior to and during the TGF-β1 treatment. Dimethyl sulfoxide (DMSO) (final concentration of 0.1%) was used as a vehicle control for these TGF-β1 inhibitors.

### 2.4. Western Blotting

Cells grown in 6-well plates were treated with TGF-β1 as described above. At 48 h post-treatment, the supernatant was removed, and the cells were washed and then lysed in 70 μL of cell lysis buffer (Cell Signaling Technology, Danvers, MA, USA). The protein concentrations of the lysates were quantified using a DC Protein Assay (Bio-Rad Laboratories, Inc., Hercules, CA, USA). The cell lysates were then loaded onto a NuPAGE 4–12% Bis-Tris protein gel (Invitrogen) and separated by electrophoresis. Following electrophoresis, the proteins were transferred to polyvinylidene fluoride membranes (Invitrogen), and nonspecific binding sites were blocked with 1% BSA in PBS with 0.1% Tween-20. The membranes were incubated together with a primary antibody (rabbit polyclonal anti-E-cadherin antibody, rabbit monoclonal anti-vimentin antibody, mouse monoclonal anti-fibronectin-EDA (Fn-EDA) antibody (Abcam, Cambridge, UK)) or rabbit monoclonal anti-phospho-Smad2 antibody (Merck, Darmstadt, Germany) at 4 °C overnight. The membranes were then incubated with horseradish peroxidase-conjugated secondary antibodies (Cell Signaling Technology) for 30 min at room temperature (RT) and visualized with a luminescent image analyzer (Image Reader LAS-4000 mini, Fujifilm, Tokyo, Japan).

### 2.5. Virus Infection

Cells were grown in 96-well plates, and, after treatment with TGF-β1, as described above, the cells were washed with PBS and incubated with the virus at a multiplicity of infection (MOI) of 1 or 5, depending on the requirements of the experiment. After a 3-h incubation period at 35 °C in a humidified 5% CO_2_ incubator with gentle shaking every 10–15 min during the first hour, the supernatant was removed, the cells were washed, and the medium was replaced with fresh medium (DMEM/2% FBS). At 24 and/or 48 hpi, the percentage of cells infected with the virus was determined by FCM analysis. The supernatants were collected at 48 hpi and then subjected to titration of viral progeny. In other experiments, a negative control using heat-inactivated RuV was also included.

### 2.6. Virus Binding Assay

The cells were seeded onto 96-well plates and then treated with TGF-β1, with or without TGF-β inhibitors, as described above. The cells were washed with ice-cold PBS and were inoculated with RuV on ice for 1 h, with gentle shaking every 10–15 min. After gently washing cells 3 times using ice-cold PBS, they were collected by trypsinization and subjected to the surface staining procedures for RuV. Then, the RuV-positive cells were determined by FCM analysis, or the cells proceeded to RNA extraction and then real-time PCR.

### 2.7. FCM Analysis

To investigate the cell surface expression of E-cadherin, vimentin and Fn-EDA proteins, after TGF-β1 treatment, the cells were stained and collected using a previously described two-step protocol for preparing adherent cells [17]. In brief, after removing the supernatant, detachment medium (DMEM containing 2.9 mM EDTA, 2% FBS) containing Live/Dead Staining Solution (Live/Dead Fixable Near-IR Dead Cell Stain Kit, Thermo Fisher Scientific, Waltham, MA, USA) and primary antibodies was added and the mixture was incubated in the incubator for approximately 1 h. After centrifugation and one wash with staining buffer (STB, cold PBS containing 5% FBS and 2 mM EDTA), the cells were incubated with a goat anti-rabbit IgG H&L (Alexa Fluor 488) secondary antibody (ab150081, Abcam) or with a fluorescein isothiocyanate (FITC)-conjugated goat anti-mouse IgG (H+L) secondary antibody (F-2761, Thermo Fisher Scientific). The cells were then washed, fixed with paraformaldehyde and subjected to FCM analysis.

For determination of the percentages of RuV-infected cells at 24 or 48 hpi, the cells were first collected and stained by incubation with detachment medium, as described above. After washing, the cells were fixed and permeabilized using a BD Cytofix/Cytoperm Fixation/Permeabilization Solution Kit (BD Biosciences, San Diego, CA, USA). The intracellular staining was performed with a mouse monoclonal anti-RuV capsid antibody (ab34749, Abcam) for 30 min at RT. The cells were then washed and incubated with a goat anti-mouse IgG H&L (Alexa Fluor 647) secondary antibody (ab150115, Abcam) solution for 30 min at RT. After washing and fixation, the cells were subjected to FCM analysis. For each sample, the data for at least 5000 gated events were collected and analyzed on a BD FACSVerse cytometer using BD FACSuite software (version 1.2; BD Biosciences). Separate negative control groups without virus inoculation as well as the cells incubated with heat-inactivated RuV were also established.

For the virus binding assay, after trypsinization and washing using STB, the cell surface staining procedures for RuV were performed on ice using the same primary and secondary antibodies as described above, for a duration of 1 h for each incubation period.

### 2.8. RNA Extraction and RT-PCR

Total RNA was extracted using TRIzol reagent (Life Technologies, Tokyo, Japan), and contaminated genomic DNA was removed by treatment with DNAse I (TaKaRa Bio, Inc., Otsu, Japan). Real-time RT-PCR was performed using a One Step TB Green PrimeScript PLUS RT-PCR Kit (Perfect Real Time) (TaKaRa Bio) in a QuantStudio3 Real-Time PCR System (Applied Biosystems, MA, USA). The following primer sequences were used: RuV, sense, 5′-CCA CTG AGA CCG GCT GCG A-3′; antisense, 5′-GCC TCG GGG AGG AAG ATG AC-3′; and PPIA, sense, 5′-ATG CTG GAC CCA ACA CAA AT-3′ and antisense, 5′-TCT TTC ACT TTG CCA AAC ACC-3′.

### 2.9. Apoptosis Assay

Tests were performed on A549 cells grown in 96-well plates using an ApoStrand ELISA apoptosis detection kit (BIOMOL, Plymouth Meeting, PA, USA). This detection system employs monoclonal antibodies to single-stranded DNA (ssDNA), which is present in apoptotic cells but not in necrotic cells or cells with DNA breaks in the absence of apoptosis. The cells were seeded at a density of 10^4^ cells/well, cultured, treated with 10 ng/mL TGF-β1 for 2 days and then infected with RuV using the same procedures as described above. ELISA was performed for apoptotic measurement at day 5 p.i. In brief, the cells were fixed for 30 min with the fixative included in the kit, as indicated by the manufacturer, and dried by incubation at 56 °C for 20 min. Formamide was then added to the cells, and they were heated at 56 °C for 30 min to denature the DNA in apoptotic cells. After blocking, the cells were incubated with an antibody mixture for 30 min, washed and then incubated with 100 μL of peroxidase substrate for 45 min. The absorbance was read using an ELISA plate reader at 405 nm. Negative controls without viral inoculation, heat-inactivated virus samples and positive controls (provided in the kit) were also included.

### 2.10. Statistical Analysis

Analysis of variance was used for statistical analysis of the results. A *p*-value < 0.05 obtained using the Tukey-Kramer test and Statcel 4 software (OMS Publishing, Inc., Tokorozawa, Saitama, Japan) was considered significant. The data are presented as the means ± SEMs.

## 3. Results

### 3.1. The EMT-Like Process of the A549 Cells Induced by TGF-β1

**Figure 1:**
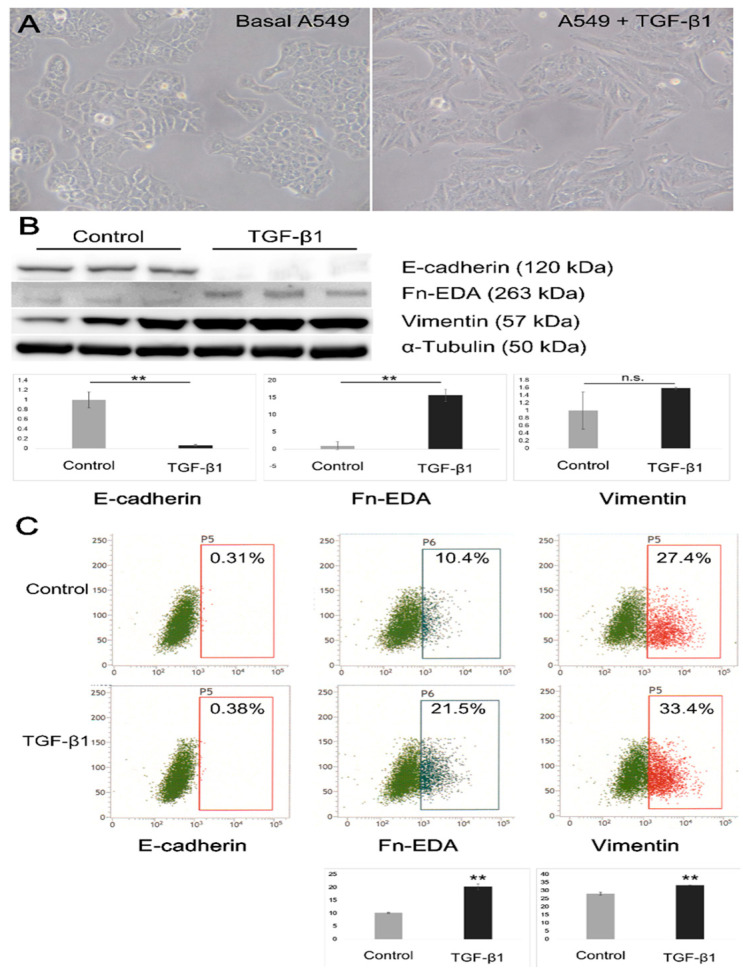
Morphological changes and expression changes in epithelial-to-mesenchymal transition (EMT)-related markers of A549 cells. (**A**) A549 cells were cultured in 6-well plates and treated with 10 ng/mL TGF-β1 in DMEM/0.1% BSA (serum-free medium) for 48 h. In the presence of TGF-β1, A549 cells changed from a pebble-like shape with clear cell–cell adhesion to a fibroblast-like spindle shape with decreased cell–cell contacts (magnification of 200×). (**B**) Western blot results show a decrease in E-cadherin expression and an increased extra domain A-containing cellular fibronectin (Fn-EDA). (**C**) Flow cytometry (FCM) analyses of cell surface expression of E-cadherin, Fn-EDA and vimentin. Control: the cells were mock-treated with serum-free medium. Images are representative of three independent experiments. **, *p* < 0.01.

In the treated A549 cells, a TGF-β1-induced EMT-like process involving morphological changes from a pebble-like shape with clear cell–cell adhesion to a fibroblast-like spindle shape with decreased cell–cell contacts was observed (Figure 1A), accompanied by a loss of E-cadherin and upregulation of extra domain A (EDA)-containing cellular fibronectin (Fn-EDA) (Figure 1B). Upregulation of vimentin was not observed by Western blot analysis; however, surface staining showed a modest increase in the percentages of A549 cells positive for vimentin, from 28.1% to 33.4%, as determined by FCM analysis (*p* < 0.01). The upregulation of Fn-EDA, from 10.1% to 20.3%, was also confirmed by FCM analysis (*p* < 0.01), as shown in Figure 1C.

### 3.2. The TGF-β1-Induced EMT-Like Process Enhances RuV Infection

**Figure 2:**
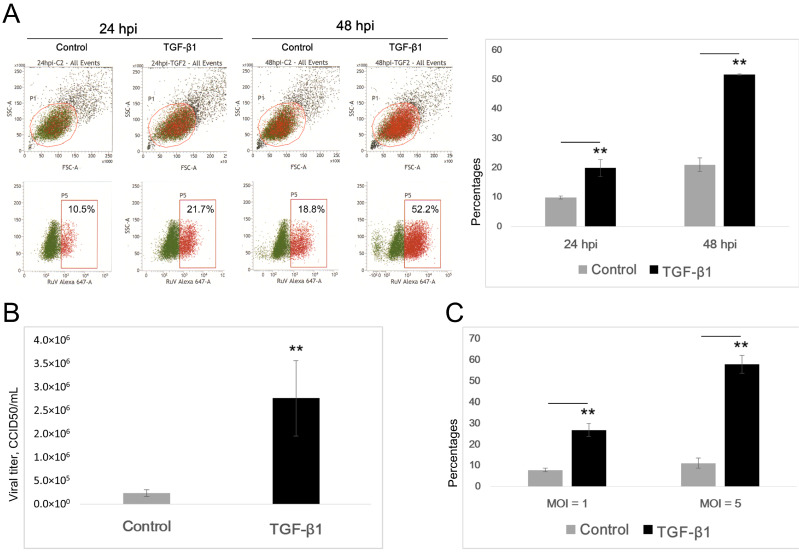
The TGF-β1-induced EMT-like process enhances the RuV infection rate in A549 cells. (**A**) Percentages of A549 cells positive for RuV determined by FCM analysis at 24 and 48 hpi. A549 cells were seeded onto 96-well plates at a density of 10^4^ cells/well and stimulated with TGF-β1 (0, 10 ng/mL) for 24 to 48 h prior to virus infection (multiplicity of infection (MOI) = 1). The cells were collected at 24 and 48 hpi and labeled with a Live/Dead Fixable Near-IR Dead Cell Stain Kit. Intracellular staining was performed with a mouse monoclonal anti-rubella virus capsid antibody followed by a goat anti-mouse IgG H&L (Alexa Fluor 647) secondary antibody. (**B**) Titers of viral progeny in the supernatants collected at 48 hpi. (**C**) Percentages of A549 cells positive for RuV after the first round of infection, at an MOI of 1 and 5, determined by FCM analysis. After the TGF-β treatment, the cells were infected with RuV at an MOI of 1 or 5, and NH4Cl (20 mM) was added at 6 hpi to prevent a second round of infection. All the above results are expressed as the mean of at least three replicates in each group, and each graph is representative of three independent experiments. **, *p* < 0.01.

The A549 cells were stimulated with 10 ng/mL TGF-β1 for 24 to 48 h prior to virus infection. We detected significant, at least twofold, increases in the percentages of TGF-β1-treated A549 cells that were positive for RuV compared with the control cells, as shown by FCM analysis at 24 and 48 hpi (Figure 2A). The above finding was supported by the CCID50 results, which showed significantly increased titers of the viral progeny produced in the supernatant collected at 48 hpi (Figure 2B).

With the addition of NH4Cl to the cell culture medium (final concentration of 20 mM) to prevent a second round of infection, the enhanced infectivity of RuV in A549 cells was strongly confirmed, with at least a threefold increase in the percentages of RuV-infected TGF-β1-treated cells compared with control cells (Figure 2C).

### 3.3. The TGF-β1-Induced EMT-Like Process Does Not Enhance Apoptosis of Infected Cells

**Figure 3:**
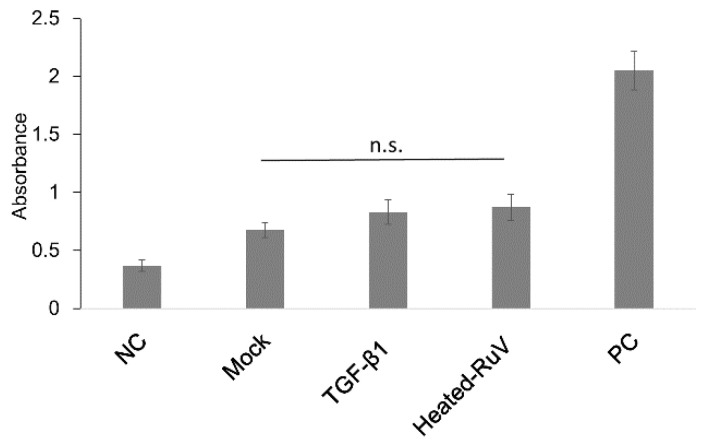
TGF-β1-induced EMT-like process does not enhance apoptosis in infected cells. ELISA detection of single-stranded DNA present in apoptotic cells at day 5 post-infection. A549 cells were seeded at a density of 10^4^ cells/well onto a 96-well flat-bottom plate, as specified by the manufacturer. A549 cells not incubated with RuV and TGF-β1-treated cells incubated with heat-inactivated RuV were used as negative controls. Single-stranded DNA provided in the ELISA kit was used as a positive control. The results are expressed as the mean of at least three replicates in each group, and the graph is representative of two independent experiments. NC, negative control; mock, cells cultured in DMEM/0.1% BSA without TGF-β1; TGF-β1, cells cultured in the presence of TGF-β1 10 ng/mL; heated-RuV, heat-inactivated RuV; PC, positive control; n.s., non-significant.

In this study, an apoptosis assay was performed on A549 cells grown in 96-well plates on day 5 p.i. As noted in Figure 3, the absorbance values of the TGF-β1-treated cells infected with RuV were not different from those of the control (mock-treated) cells or the TGF-β1-treated cells incubated with heat-inactivated virus. The above results imply that no significant apoptosis occurred in either of the RuV-infected groups (mock- or TGF-β1-treated) until day 5 p.i.

### 3.4. The TGF-β1-Induced EMT-Like Process Enhances Rubella Virus Binding to A549 Cells as Shown by the Virus Binding Assay

**Figure 4:**
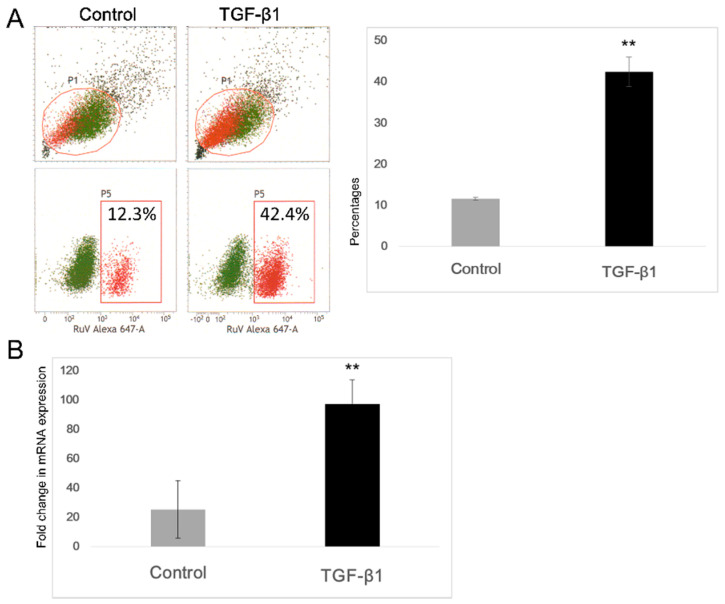
Virus binding assay showed an enhancement in rubella virus binding to the TGF-β-treated A549 cells. After TGF-β1 treatment, the cells were inoculated with RuV at an MOI of 5 on ice for 1 h. After washing, the surface staining procedures for RuV were conducted, and the cells were either analyzed by FCM or were subjected to RNA extraction and then real-time PCR using a delta-delta Ct method. (**A**) Percentages of RuV-positive cells determined by FCM analyses. Values are means ± SEM. (**B**) Increased viral RNA expression in the cells treated with TGF-β1. The data shown are the means of the fold change of relative expression values (normalized with the internal control PPIA gene expression) ± SEM. All the results are expressed as the mean of at least three replicates in each group, and each graph is representative of three independent experiments. **, *p* < 0.01.

After the TGF-β1 treatment step, the cells were inoculated with RuV on ice for 1 h. After washing, the cells were subjected to the surface staining procedures for RuV or to RNA extraction followed by real-time PCR. Results obtained by FCM analysis showed that the percentages of RuV-positive cells were significantly increased, at least threefold, in the TGF-β1-treated cells compared with the control cells. The above finding was supported by the real-time PCR results using the delta-delta Ct method, showing a corresponding increase in RuV RNA expression, after normalization with the internal control peptidylprolyl isomerase (PPIA) gene expression (Figure 4).

### 3.5. The TGF-β1-Induced EMT-Like Process Enhances Virus Binding and Infection in A549 Cells via the Smad Pathway

To investigate the role of TGF-β1 in the enhancement of RuV binding and infection in A549 cells, various TGF-β1 inhibitors were used to suppress the TGF-β1 downstream pathways, including p38 MARK, MEK/ERK1/2 and SMAD2/3, as well as the JNK pathway.

**Figure 5:**
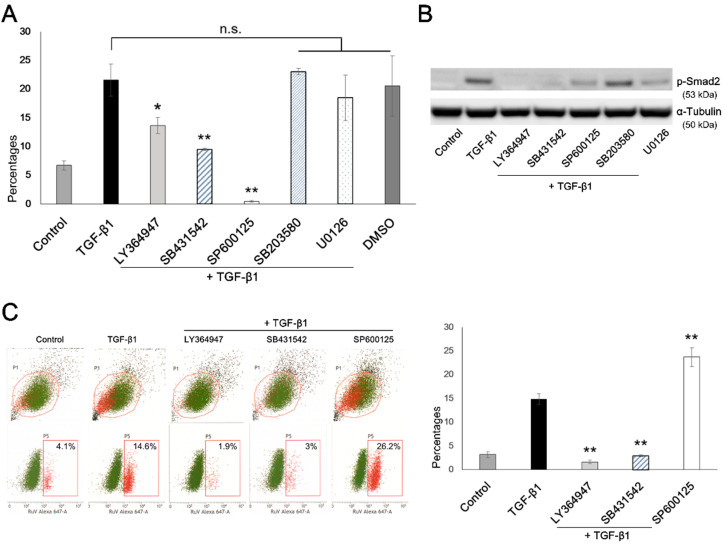
The TGF-β1-induced EMT-like process enhances RuV binding and infection via Smad signaling. (**A**) Percentages of RuV-infected cells after pretreatment with TGF-β inhibitors. The cells were incubated with TGF-β inhibitors for 1 h prior to and during the TGF-β1 treatment. After washing, the cells were infected with RuV at an MOI of 1 for 3 h in the incubator, and medium containing NH4Cl (final concentration 20 mM) was added at 6 hpi. The cells were collected and subjected to intracellular staining to detect the presence of the RuV capsid protein, as described previously. (**B**) Western blot results show changes in the p-Smad2 expression in the presence of the used TGF-β inhibitors. (**C**) Percentages of RuV-positive cells determined by FCM analysis, obtained by the virus binding assay following pretreatment with TGF-β inhibitors LY364947, SB431542 and SP600125. The A549 cells treated with one of the three above inhibitors and TGF-β1 proceeded to virus infection (MOI = 1) for 1 h on ice. The cell surface staining for RuV was performed as described earlier, and the percentages of RuV-positive cells were determined by FCM analysis. All the results are expressed as the mean of at least three replicates in each group ± SEM, and each graph is representative of three independent experiments. *, *p* < 0.05; **, *p* < 0.01; n.s., non-significant.

The results of FCM analysis of the cells collected during the post-infection period showed that no suppressive effects were noted with SB203580 or U0126, inhibitors of p38 MARK and MEK/ERK1/2 signaling, respectively, while almost no RuV infection was observed in the A549 cells after treatment with SP600125, an inhibitor of JNK signaling. Interestingly, LY364947 and SB431542, inhibitors of the TGF-β1/Smad signaling, displayed an appropriate inhibitory effect on the enhanced infectivity of RuV by TGF-β1, with the corresponding percentages of RuV-infected cells returning close to the levels as seen in the control cells (*p* < 0.05 and *p* < 0.01, respectively) (Figure 5A).

The above results were further confirmed with the virus binding assay results, showing that the percentage of RuV-binding cells in the cells treated with TGF-β1 and in the presence of LY364947 or SB431542 was found to be almost equal to that observed in the control cells (*p* < 0.01). Meanwhile, SP600125 treatment did not result in the suppression of virus binding but enhanced the percentages of RuV-positive cells (*p* < 0.01) (Figure 5C). In concordance with the changes in virus binding, the Western blot results showed no or very little expression of p-Smad2 protein in the presence of the Smad signaling inhibitors, LY364947 and SB431542 (Figure 5B).

## 4. Discussion

TGF-β is a pleiotropic cytokine that carries out functions such as cellular differentiation, proliferation, migration, adhesion, extracellular matrix (ECM) synthesis, apoptosis and cancerogenesis. TGF-β signaling is indispensable in lung development and in physiology, as well as in the pathogenesis of pulmonary diseases [18]. TGF-β is expressed by multiple cell types, including epithelial cells, fibroblasts and macrophages. TGF-β1 levels are elevated in the airways of humans with chronic respiratory diseases, such as asthma, IPF and COPD, as well as tobacco smokers [19,20,21]. TGF-β1 is a major inducer of EMT, and increased EMT has been reported in patients with the abovementioned chronic lung diseases [11,12,13,22].

In in vitro experiments, the TGF-β1-induced EMT model for lung epithelial cells has been well established using A549 cells [14,23]. As noted in Figure 1, the A549 cells used in this study did not express E-cadherin on their surface, in accordance with the cell bank’s notice at the time of purchase. Although upregulation of vimentin abundance was not observed by Western blot analysis, which was consistent with the findings of a previous study [14], evidence of the TGF-β1-induced EMT-like process performed in A549 cells was obtained in the form of the upregulation of both cell surface markers of the mesenchymal characteristics, vimentin (although modest) and Fn-EDA, as observed by FCM analysis. This was supported by a downregulation of E-cadherin and upregulation of Fn-EDA, as shown by Western blot analysis.

To investigate the influence of the above experimental EMT process on RuV infection, we obtained conclusive results showing that the TGF-β1-induced EMT-like process enhanced virus binding and infection at least threefold more than that in the control cells, as determined by FCM analysis, titration of the collected supernatants, as well as real-time PCR. However, this effect of enhanced infectivity in RuV caused no significant changes in the apoptosis of the infected cells until day 5 p.i. It has been well established that apoptosis is an important immune response for the control of viral infections. The induction of apoptosis during RuV infection varies among cell types, and, in most cases of induced apoptosis, viral protein synthesis and virion release occur well in advance of extensive apoptosis (reviewed in refs) [24]. The above finding supports the current understanding of RuV as being able to suppress apoptosis through its capsid protein [25].

With the use of various TGF-β inhibitors before TGF-β1 treatment and virus infection, we found that LY364947 and SB431542, inhibitors of TGF-β1/Smad signaling, could inhibit the enhancement of RuV infectivity in the TGF-β1-treated A549 cells. LY364947 is a selective inhibitor targeting type 1 TGF-β receptors (also known as ALK5, activin receptor-like kinase receptor 5), while the widely used SB431542 blocks several ALKs, including ALK4, ALK5 and ALK7 [26,27,28]. This finding was confirmed by the virus binding assay results, showing that, in the presence of LY364947 or SB431542, the percentages of RuV-positive cells decreased to the levels among those of the control cells (Figure 5).

For inhibition of the p38 MAKP and MEK/ERK1/2 signaling pathways, SB203580 and U0126, respectively, showed no suppressive effect on the enhanced RuV infection caused by the TGF-β1-induced EMT-like process. In addition, it was found that the JNK signaling is unlikely to be responsible for such an enhancement effect, as the presence of its inhibitor, SP600125, in the TGF-β1 treatment resulted in unsuccessful infection of RuV in A549 cells, with the percentages of RuV-positive cells decreasing to almost 0%, while an increase in virus binding was observed (Figure 5). A previous study suggested that inhibition of JNK results in the enhancement of TGF-β1-activated Smad2 signaling [29]. Therefore, the above phenomenon could be explained as follows: with the inhibition of the JNK pathway alone, enhanced virus binding caused by the TGF-β1-activated Smad2 signaling still occurred, but the internalization and/or transcription/replication of the virus were completely suppressed as the overall protein synthesis was repressed as a result of the inhibited JNK signaling [30].

It has been well established that, in addition to its role in EMT, TGF-β1 induces extracellular matrix protein deposition [31]. The findings of this study suggest that cell surface marker changes during EMT reprograming, as well as changes in ECM proteins, might facilitate virus binding and infection in A549 cells.

In conclusion, this study suggests that the TGF-β1-induced EMT-like process enhances rubella virus binding and infection in A549 cells via the Smad pathway. Further studies are necessary to identify possible proteins that facilitate viral binding and entry into treated cells.

## Data Availability

No new data were created or analyzed in this study.
